# Effect of umbilical cord blood-mononuclear cells on knee osteoarthritis in rabbits

**DOI:** 10.1186/s13018-024-04815-8

**Published:** 2024-05-30

**Authors:** Yuhang Fu, Chi Zhang, Yong Yang, Baisui Zhou, Meng Yang, Guoshuai Zhu, Yonglin Zhu

**Affiliations:** 1 The Second School of Clinical Medicine of Binzhou Medical University, Yantai, 264199 Shandong Province China; 2 Yantai Affiliated Hospital of Binzhou Medical University, Yantai, 264199 Shandong Province China; 3 Yantai City Yantai Mountain Hospital, Yantai, 264008 Shandong Province China

**Keywords:** Osteoarthritis, Umbilical cord blood-mononuclear cells, Platelet-rich plasma

## Abstract

**Background:**

To investigate the effect and underlying mechanism of umbilical cord blood-mononuclear cells (UCB-MNCs) in treating knee osteoarthritis (KOA) in rabbits.

**Methods:**

A rabbit KOA model was prepared by anterior cruciate ligament transection (ACLT). Fifty New Zealand white rabbits were randomly divided into the control group, model group, sodium hyaluronate (SH) group, platelet-rich plasma (PRP) group and UCB-MNC group. Knee injections were performed once a week for five consecutive weeks. The gross view of the knee joint, morphology of knee cartilage and structural changes in the knee joint were observed on CT scans, and graded by the Lequesne MG behavioral score and the Mankin score. TNF-α and IL-1β levels in the synovial fluid of the knee were measured by the enzyme-linked immunosorbent assay (ELISA). Expression levels of MMP-13 and COL-II in the knee cartilage were detected by Western blotting and qRT-PCR.

**Results:**

The Lequesne MG behavioral score and the Mankin score were significantly higher in the model group than those in the control group (*P* < 0.05). Rabbits in the SH, PRP and UCB-MNC groups had sequentially lower scores than those in the model group. Imaging features of KOA were more pronounced in the model group than in the remaining groups. CB-MNC significantly relieved KOA, compared to SH and PRP. Significantly higher levels of TNF-α and IL-1β in the synovial fluid of the knee, and up-regulated MMP-13 and down-regulated COL-II in the knee cartilage were detected in the model group than in the control group. These changes were significantly reversed by the treatment with SH, PRP and UCB-MNCs, especially UCB-MNCs.

**Conclusion:**

Injections of UCB-MNCs into knees protect the articular cartilage and hinder the progression of KOA in rabbits by improving the local microenvironment at knee joints.

## Introduction

Knee osteoarthritis (KOA) is a kind of degenerative disease characterized by progressive breakdown of articular cartilage, local pain, and limited range of motion [1, 2]. Oral medications (e.g., nonsteroidal anti-inflammatory drugs, opioids, and symptomatic slow-acting drugs), injections of sodium hyaluronate (SH) or platelet-rich plasma (PRP) into knees, and surgical procedures are currently the major therapeutic strategies for KOA. Surgery is usually the last resort for severe KOA [3], posing a great financial burden on affected patients [4]. Cheap, effective, and safe treatment approaches of KOA are urgently needed to promote cartilage regeneration and inhibit local inflammation [5].

Stem cells have been extensively applied for spinal cord damage and neonatal hypoxic-ischemic encephalopathy, owing to their regenerative and anti-inflammatory properties [6]. Human umbilical cord blood (hUCB)-stem cells present high expansion potential, low immunogenicity and can be simply isolated and cultured [7–9]. Human umbilical cord blood-mononuclear cells (hUCB-MNCs) consist of hemopoietic stem cells (HSCs), mesenchymal stem cells (MSCs), endothelial progenitor cells (EPCs), lymphocytes and monocytes. They demonstrate strong tissue-repairing and anti-inflammatory functions [10]. Preclinical evidences have validated the high efficacy of MSC injections in accelerating cartilage repair in small animals with OA [11, 12]. hUCB-MNCs have also been reported to regulate immune responses in inflammatory-related diseases, such as renal tubulointerstitial fibrosis [13] and lipopolysaccharide-induced acute kidney injury [14]. However, hUCB-MNC has never been employed in the treatment of KOA. Given the advantages of cheapness, low immunogenicity and nothingness with ethical issues, it is postulated that hUCB-MNC may be a promising treatment for KOA. Therefore, we conducted the present experiment to explore the effect of hUCB-MNC on KOA in rabbits.

## Materials and methods

### A rabbit model of KOA

**Fig. 1:**
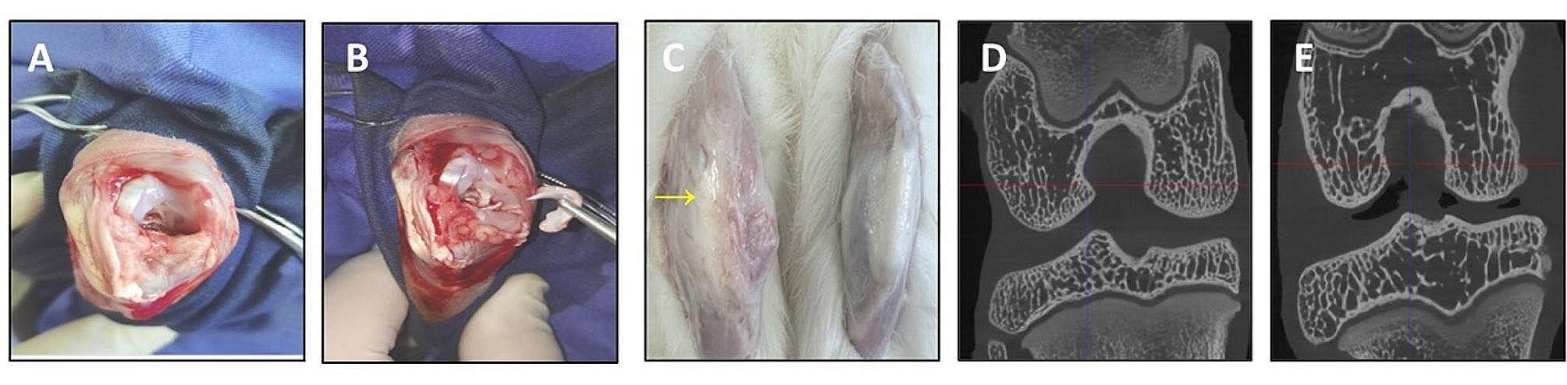
Diagram of creating the KOA rabbit model. (**A**) Exposure of the knee joint. (**B**) Transection of the anterior cruciate ligament and excision of the medial meniscus. (**C**) Visible swelling of the knee (arrow point). (**D**) A micro-CT scan visualizing the formation of a bone capsule

Fifty male New Zealand white rabbits aged 6 months and weighing 2.5–3.0 kg (Xilingjiao Breeding Centre, Jinan, China; license number: SCXK (Lu) 20,180,010) were habituated for one week. They were randomly divided into the control group, model group, SH group, PRP group and hUCB-MNC group, with 10 rabbits per group. The rabbit KOA model in the latter four groups was prepared by the anterior cruciate ligament transection (ACLT) as previously reported [15]. In brief, a median incision 4 cm in length was made on the knee joint to expose the joint cavity via a medial parapatellar approach. External rotation of the patella, transection of the anterior cruciate ligament and excision of the medial meniscus were then performed. A daily intramuscular injection of 8.0 × 10^5^ units of penicillin was given for three days to prevent postoperative infections. One week after surgery, rabbits were assisted to make early activities for half an hour once and twice a day. Six weeks after surgery, the KOA rabbit model was established [16] (Fig. 1). All animal experiments were conducted under the Guidelines for Humanitarian Treatment of Laboratory Animals issued by the Ministry of Science and Technology of China in 2006, and approved by the Animal Care and Use Committee of Binzhou Medical College.

### Animal interventions

hUCB-MNCs and PRP were provided by the Cord Blood Hematopoietic Stem Cell Bank of Shandong Province. Briefly, hUCB-MNCs (0.5 × 10^8^/4 ml) cryopreserved in liquid nitrogen were resuscitated in a water bath at 37℃. Cells suspended in the washing solution (normal saline + 20% human albumin) in a 3:1 ratio were centrifuged at 1200 r for 10 min. The precipitant was re-suspended in the washing solution and centrifuged again. Finally, a single-cell suspension of hUCB-MNCs (4 × 10^6^ cells/0.3 ml) was prepared. PRP (2.5 × 10^9^/4 ml) was centrifuged at 1200 r for 10 min and prepared at a platelet concentration of 4.5 × 10^8^ cells/0.3 ml. Then, 0.3 ml of normal saline, 0.3 ml of sodium hyaluronate (20 mg/2 ml), 0.3 ml of PRP and 0.3 ml of cell suspension containing 4 × 10^6^ hUCB-MNCs were injected into knees. from the 7th week after surgery once a week for five weeks in the model group, SH group, PRP group and hUCB-MNC group, respectively. Rabbits in the control group were treated with blank control.

### Behavioral assessments

Behavioral assessments were carried out by grading the Lequesne MG behavioral score as previously reported [17]. On the 7th day after the last intervention, it was administered in a double-blind fashion by two investigators who were not involved in the experiment but familiar with the behavioral assessment.

### Micro-CT

High-resolution micro-CT scans (25 μm, 80 kV) of the right knee joint of the rabbit were obtained using a small animal CT system (Hiscan).

### Pathological staining

After five weeks of injections, rabbits were sacrificed to collect the cartilage specimens of femoral condyles on the modeling side, which were fixed in 10% paraformaldehyde for 48 h, decalcified with 10% EDTA for 8 wk, dehydrated, and paraffin-embedded into 5-μm -thick sections. After permeabilization in xylene and deparaffinization, cartilage specimens were stained with hematoxylin and eosin (H&E) staining solution and Safranin O/ Fast green stain (Solarbio, Beijing, China). The morphology of the cartilage tissues in three randomly selected fields per sample was observed under an inverted microscope, and the damage to the cartilages was quantified using the Mankin scoring system as follows [18].

### ELISA

After five weeks of drug intervention, the animals were sacrificed by air occlusion. The hair around the knee joint was shaved, and the skin anterior to the knee joint was cut along the midline, meanwhile preserving the joint capsule. Every 2 ml of sterile saline was aspirated with a 5 ml syringe and slowly injected into the joint cavity along the articular surface of the patella. Joint fluid was extracted from sites as far as possible, and the supernatant was subsequently retained by centrifugation and stored at -80℃ for further analysis. The concentrations of tumor necrosis factor-α (TNF-α) and interleukin-1β (IL-1β) in joint fluid were detected by rabbit specific ELISA kit.

### qRT-PCR

Total RNAs in the articular cartilages were extracted using the TRIzol method (Takara, Beijing, China), and the concentration and purity of RNAs were determined using a spectrophotometer (Thermo Scientific, MA, U.S.A.). The extracted RNAs were reversely transcribed into cDNAs. Subsequently, amplification reactions of qRT-PCR using the cDNA as a template were performed on the fluorescence quantitative PCR system (Applied Biosystems, MA, U.S.A.). Relative levels were normalized to that of GAPDH using the 2^−ΔΔCT^. Sequences of primers (Accurate Biotechnology, Changsha, China) used in qRT-PCR are listed in Table 1.

**Table 1:** Sequences of primers used in qRT-PCR

Primers	Forward/Reverse	Sequence (5’→3’)
GAPDH	Forward	TCCTGCACCACCAACTGCTTA
	Reverse	GGTCTTCTGGGTGGCAGTGAT
MMP13	Forward	GGTCTTCTGGCTCACGCTTTTC
	Reverse	ATGGGCAGCAACGAGAAACAAG
COL-II	Forward	GTGGTGACAAAGGCGAAAAGG
	Reverse	CCAGCCTTCTCGTCAAATCCTC

### Western blotting

Total proteins were extracted from knee cartilage tissues using the RIPA lysate (Meilunbio, Dalian, China). Protein samples were loaded onto SDS-PAGE gel (Beyotime, Shanghai, China) for electrophoresis and transferred onto a PVDF membrane. After immersing in 5% skim milk for 2 h, membranes in interested sizes were incubated with the primary antibodies of anti-β-actin (1:1000, Sc-47,778, Santa Cruz Biotechnology, TX, U.S.A.), anti-MMP-13 (1:1000, 18165-1-AP, Proteintech, CA, U.S.A.), anti-Col-II (1:1000, 28459-1-AP, Proteintech), anti-AKT1/2/3 (1:1000, bs- 6951R, Bioss, MA, U.S.A.), anti-phospho-AKT (1:1000, bs0876R, Bioss), anti-PI3KCA (1:1000, 2067R, Bioss) and anti-PI3K p110 beta (1:1000, bs-10657R, Bioss) overnight at 4 °C. On the other day, they were incubated with HRP-linked anti-rabbit IgG (1:5000, #7074, Cell Signaling Technology, MA, U.S.A.) and HRP-linked anti-mouse IgG (1:5000, #7076, Cell Signaling Technology) at room temperature for 1 h. Images were visualized using an enhanced chemiluminescence kit (Meilunbio) and grey values were analyzed using ImageJ software.

### Statistical analysis

Statistical analysis was performed using SPSS statistical software. Measurement data were expressed as mean ± standard deviation ($\stackrel{-}{\varvec{x}}$±s), and differences among three and more groups were analyzed by one-way ANOVA. A significant difference was determined by a *p* value of less than 0.05.

## Results

### Behavioral changes in KOA rabbits

**Fig. 2:**
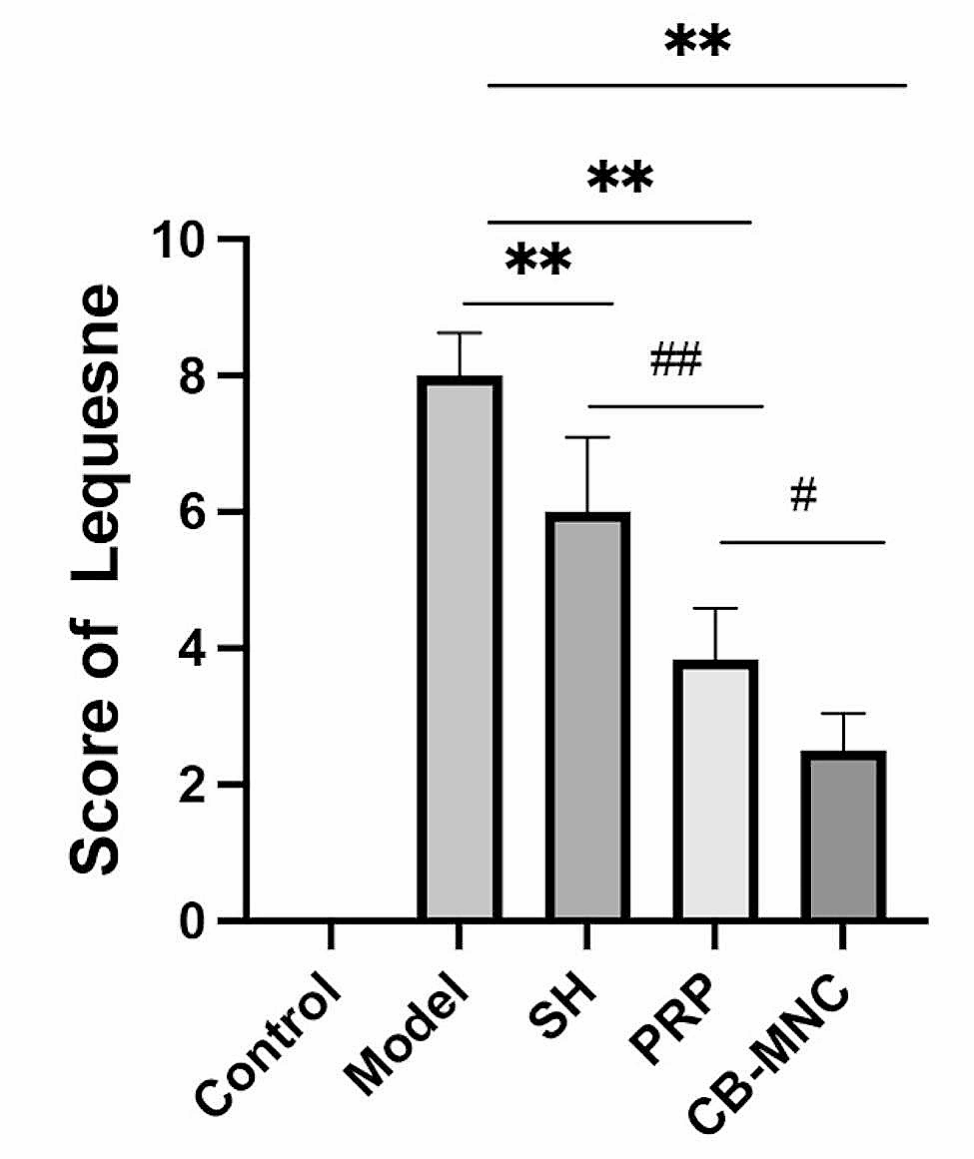
Lequesne MG behavioral scores *n* = 6. ^#^*P* < 0.05 and ^##^*P* < 0.01 vs. UCB-MNC group; ^*^P < 0.05 and ^**^P < 0.01 vs. model group

Behavioral changes in rabbits were assessed using the Lequesne MG behavioral scoring system. Rabbits in the control group were graded with 0 points, indicating no behavioral changes. The Lequesne MG behavioral scores in the model group, SH group, PRP group and UCB-MNC group were 8.00 ± 0.58 points, 6.00 ± 1.00 points, 3.80 ± 0.69 points and 2.50 ± 0.50 points, respectively. It is suggested that injections of CB-MNCs into knees significantly improve animal behavior, and this protective efficacy was superior to those of SH and PRP (Fig. 2).

### Gross morphology of knee cartilages

**Fig. 3:**
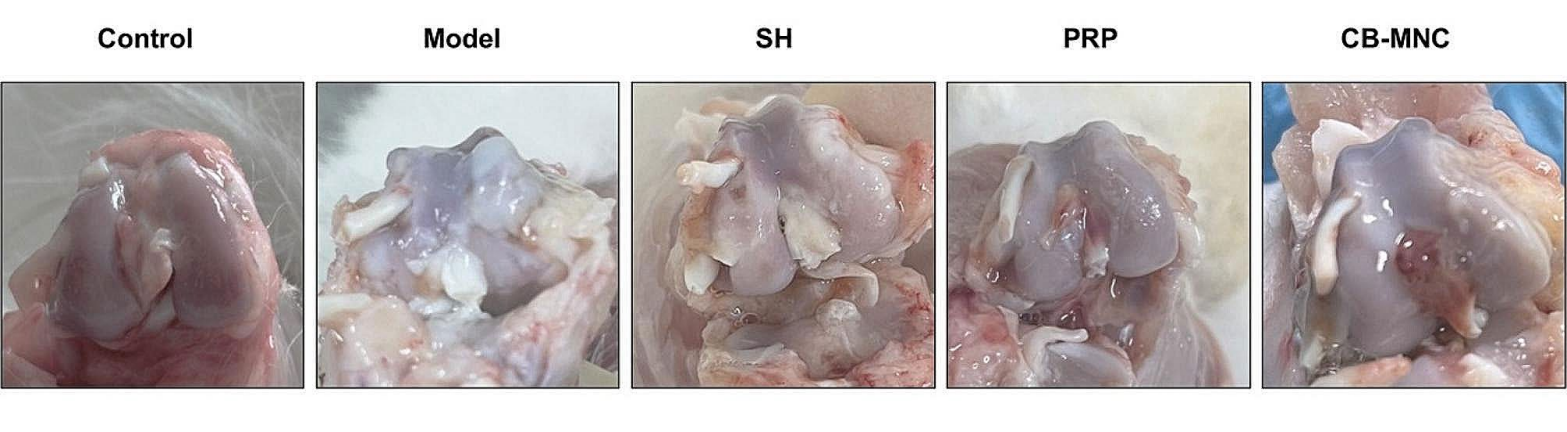
Gross views of the cartilages of femoral condyles

The gross view of the cartilage of femoral condyle was observed. Rabbits in the control group presented bright and translucent cartilages, as well as smooth surfaces of knees. Significant changes in the knee joint were observed in the model group, including rough and uneven cartilages and irregular osteophytes formed on the knee edge. Similar morphological manifestations of rough and uneven cartilages and formation of osteophytes were found in the SH group. In the PRP group, superficial cracks were occasionally observed on the surface of cartilages. KOA-induced morphological changes were significantly alleviated in the UCB-MNC group, manifesting as sporadic rough cartilages and regular edges of knees (Fig. 3).

### Micro-CT scanning of knees

**Fig. 4:**
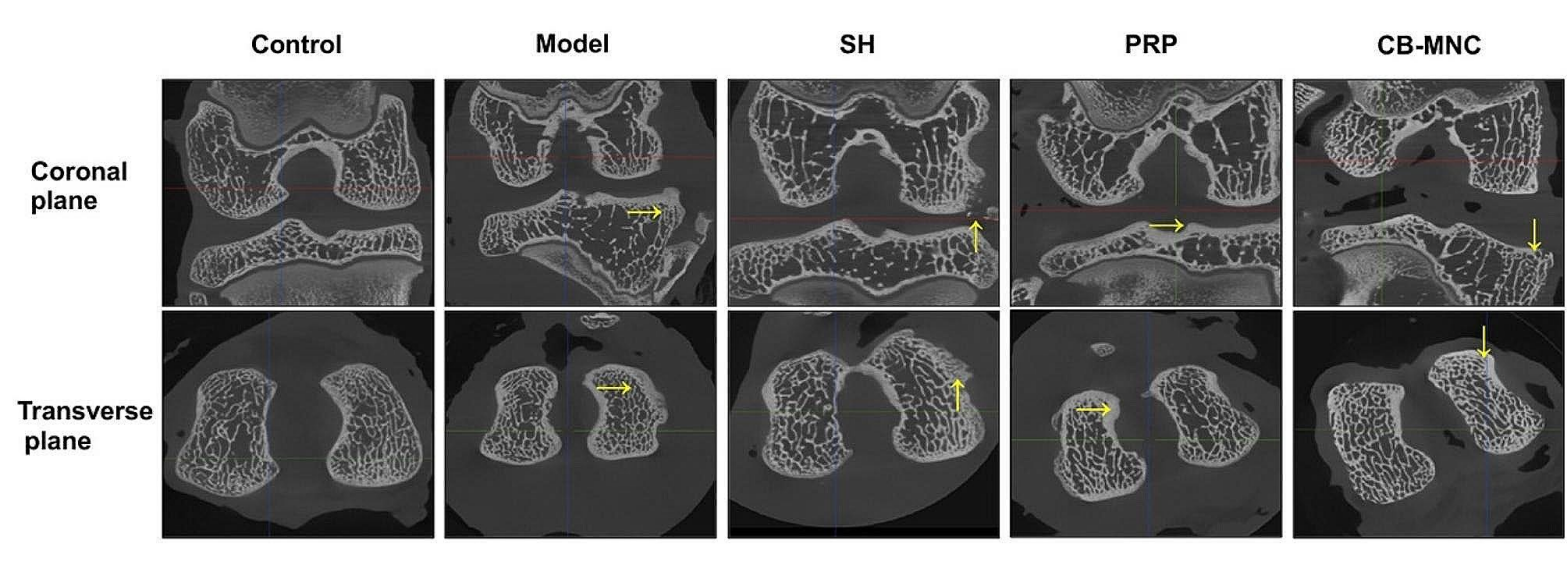
Coronal and cross-sectional micro-CT scans of knees in control group, model group, SH group, PRP group and UCB-MNC group. *n* = 3. Pathological changes were marked by arrows

In the control group, micro-CT scans showed smooth articular surfaces on the femoral condyles and tibial plateaus of knee joints, with normal joint spaces but no bone hyperplasia or osteoporosis. Significant narrowing of joint space and formation of osteophytes were observed in the model group and SH group. Injections of PRP and UCB-MNCs into knees effectively widened joint space and reduced osteophytes. Overall, micro-CT scanning revealed a protective effect of injections of PRP and UCB-MNCs against KOA-induced articular injuries (Fig. 4).

### Cartilage destructions assessed by the Mankin scoring system

**Fig. 5:**
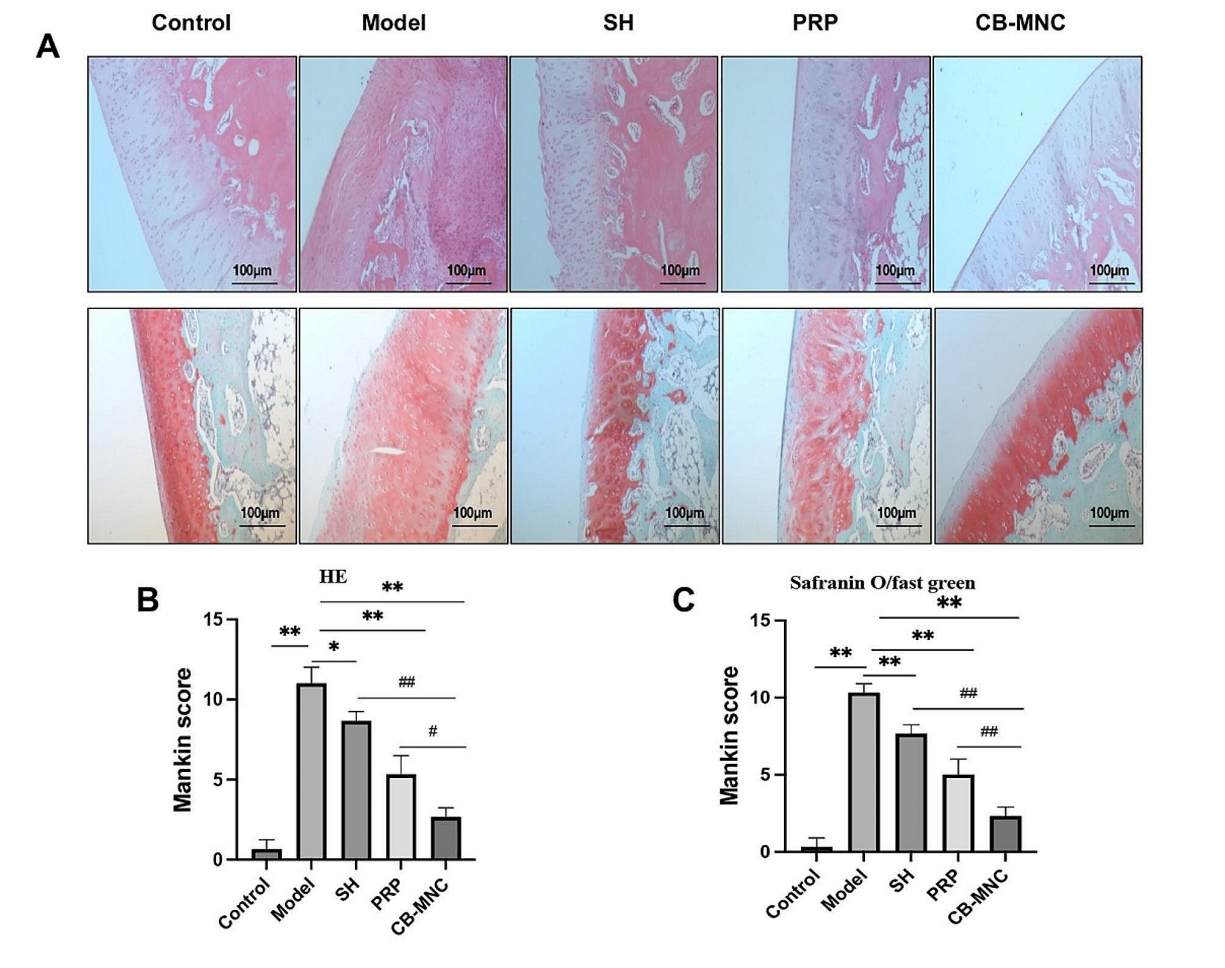
**A** Representative images of H&E staining and Safranin O/fast green staining for articular cartilages; **B** Mankin score of H&E; **C** Mankin score of safranin O/fast green, *n* = 3. ^#^*P* < 0.05 and ^##^*P* < 0.01 vs. UCB-MNC group; ^*^P < 0.05 and ^**^P < 0.01 vs. model group

H&E and Safranin O/ Fast green staining of the articular cartilages of femoral condyles on the right side were performed via the Mankin scoring system. Well-organized articular chondrocytes with uniform nuclei and chromatin were observed in the control group. However, clumped clustering of articular chondrocytes with a disordered arrangement, pyknosis and presence of cartilaginous frailties indicated severe KOA in the model group. The surface layer of the cartilage in the SH group exhibited a concave morphology or small cracks, while the chondrocytes appeared to be disorganized. Injections of PRP into knees protected against KOA-induced articular cartilage injuries, as shown by a relative smooth surface of the cartilage, although chondrocytes were disorganized and minor fissures extended into the cartilage layer. A smooth surface of chondrocytes in arrangement was observed in the UCB-MNC group (Fig. 5A). Consistently, the Mankin score was significantly higher in the model group than in the control group, and dropped in the SH group, PRP group, and especially the UCB-MNC group (Fig. 5B, C).

### Inflammatory response in KOA rabbits

**Fig. 6:**
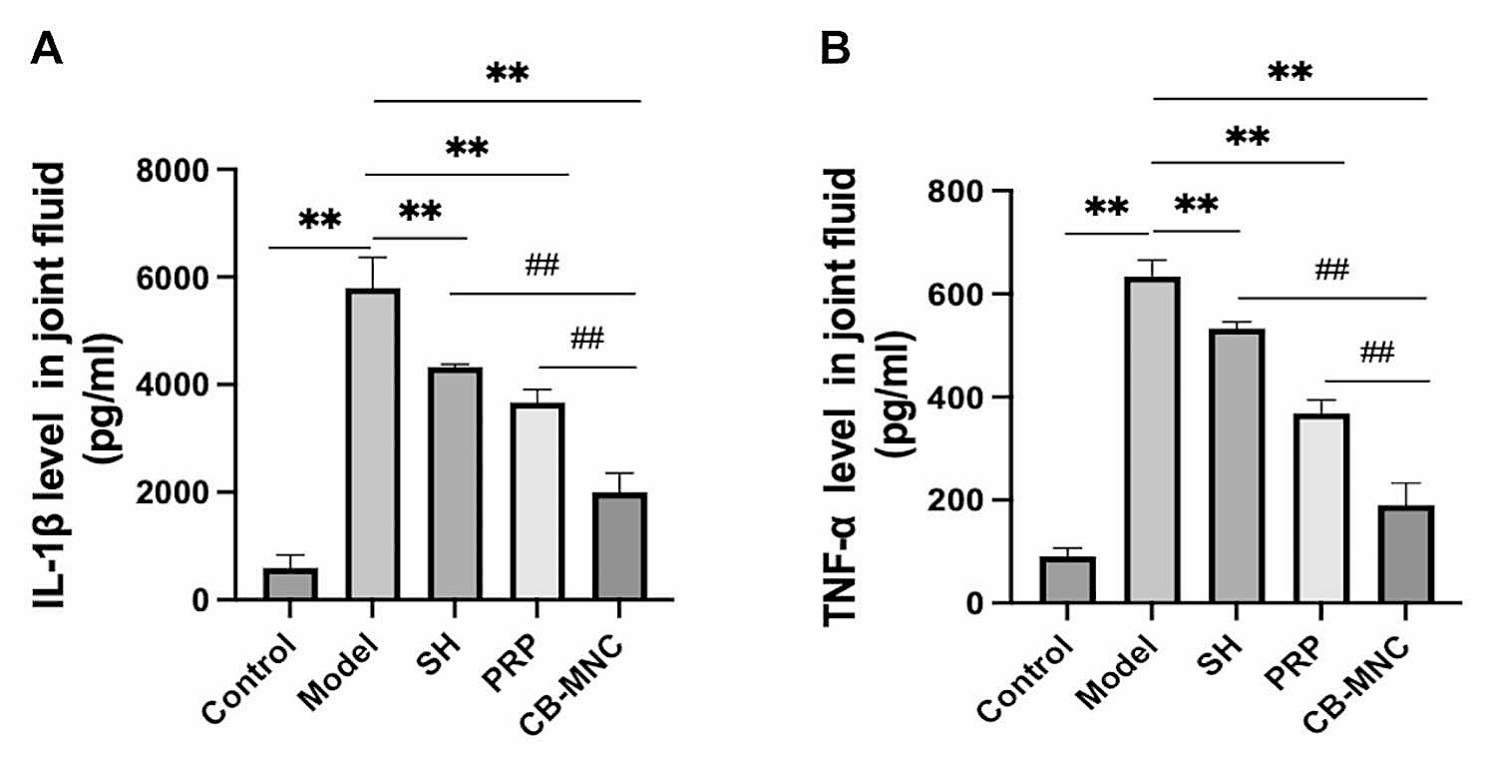
Relative levels of IL-1β (**A**) and TNF-α (**B**) in the synovial fluid of knees in the control group, model group, SH group, PRP group and UCB-MNC group. *n* = 10. ^#^*P* < 0.05 and ^##^*P* < 0.01 vs. UCB-MNC group; ^*^P < 0.05 and ^**^P < 0.01 vs. model group

IL-1β and TNF-α levels in synovial fluid of knees were measured by ELISA. They were significantly higher in the model group than in the control group (*P* < 0.01). IL-1β and TNF-α levels were significantly lower in the SH group, PRP group and UCB-MNC group than in the model group, and the most pronounced reduction was detected in the UCB-MCN group (*P* < 0.01, Fig. 6). It is suggested that injections of UCB-MNCs into knees effectively inhibited local inflammatory response in KOA rabbits.

### mRNA levels of MMP-13 and COL-II in rabbit cartilages

**Fig. 7:**
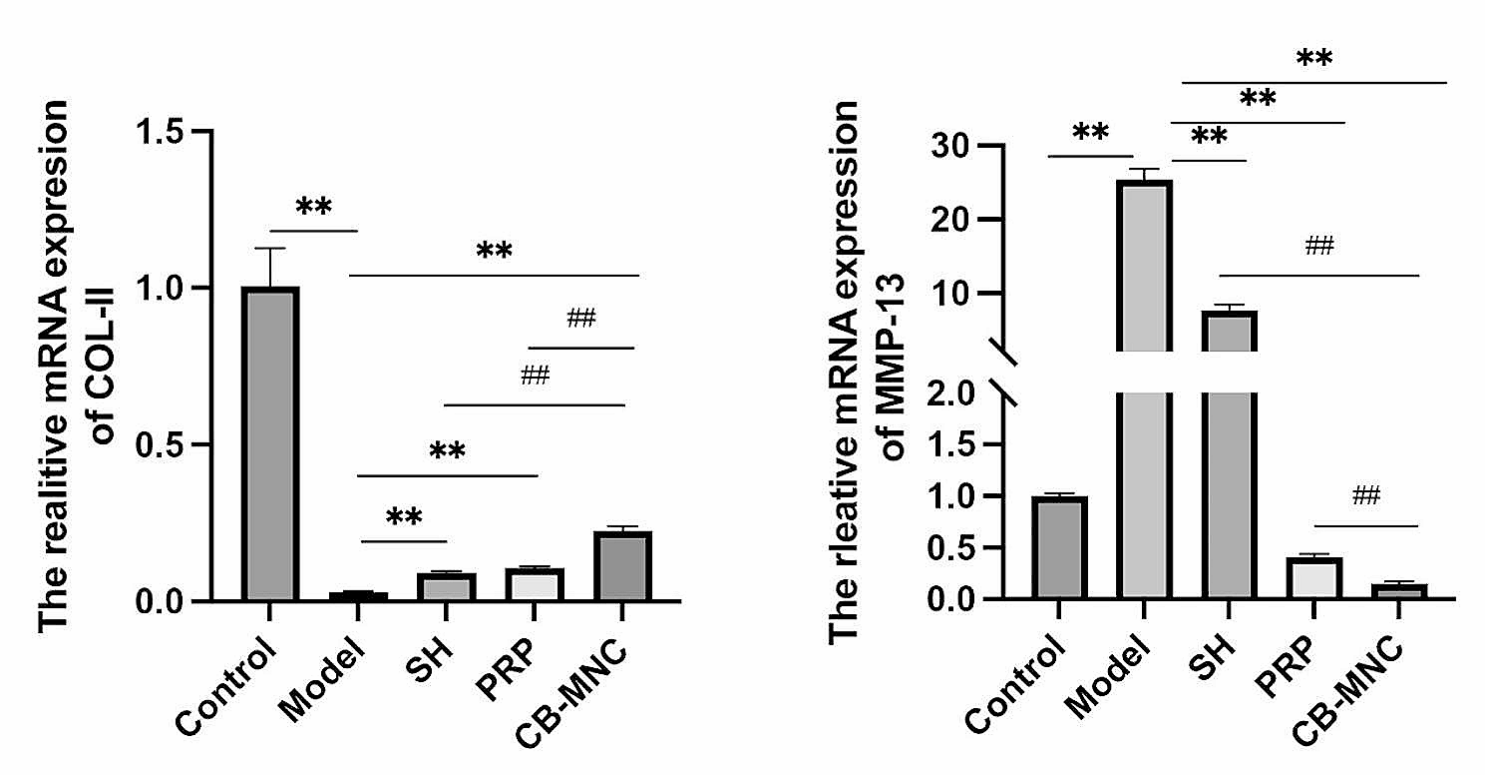
mRNA levels of MMP-13 (**A**) and COL-II (**B**) in cartilage tissues in the control group, model group, SH group, PRP group and UCB-MNC group. *n* = 6. ^#^*P* < 0.05 and ^##^*P* < 0.01 vs. UCB-MNC group; ^*^P < 0.05 and ^**^P < 0.01 vs. model group

Compared with those in the control group, the mRNA level of MMP13 was significantly upregulated and that of COL-II downregulated in the cartilage tissues of the model group. Injections of SH, PRP and UCB-MNCs into knees all significantly downregulated MMP13 and upregulated COL-II in cartilage tissues, especially in the latter group (Fig. 7).

### Protein levels of MMP-13, COL-II, and activation of PI3K/AKT signal pathway in rabbit cartilage

**Fig. 8:**
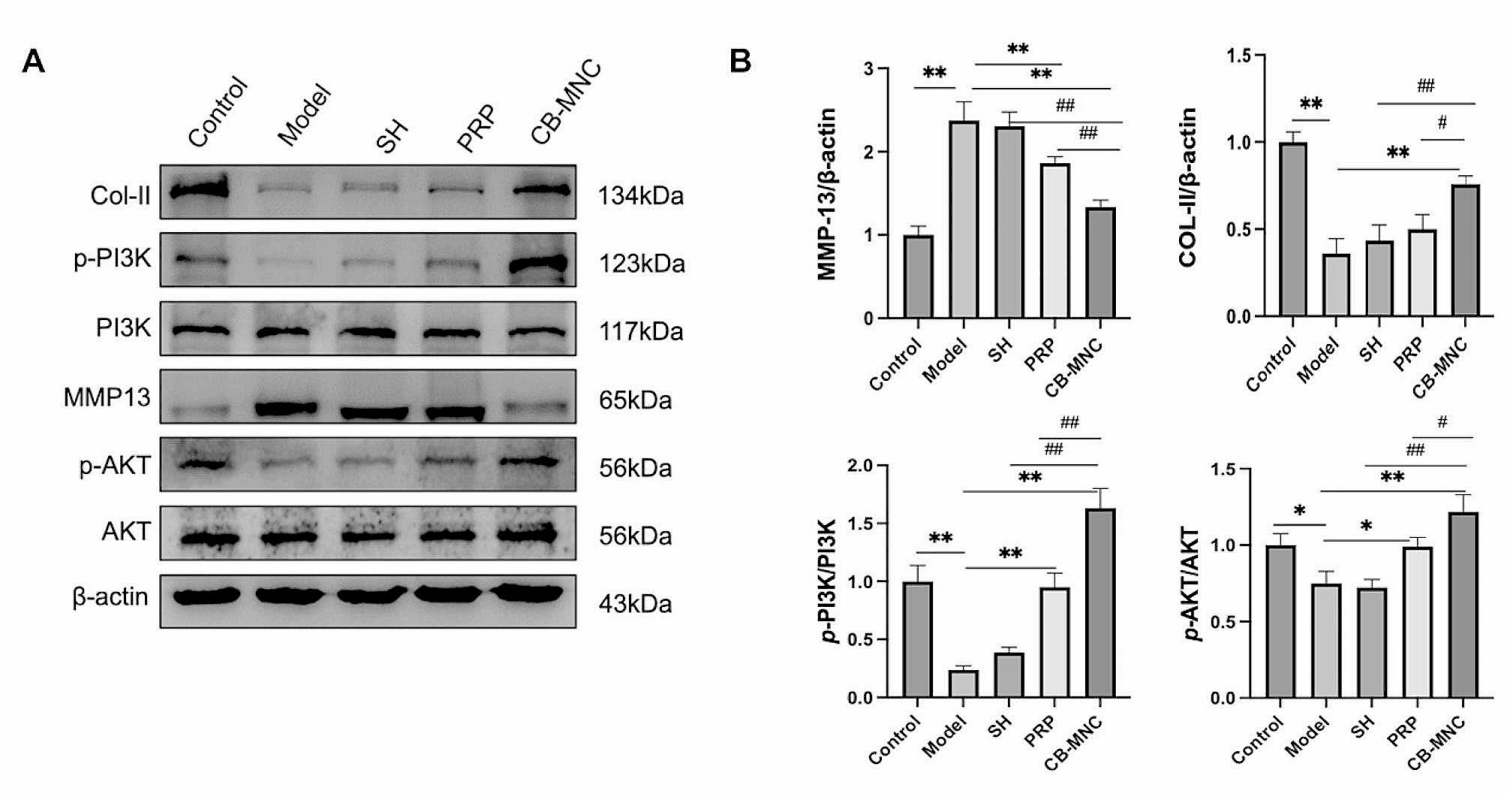
Protein levels of MMP-13, COL-II, p-PI3K and p-AKT protein (**A**), and their quantitative analyses (**B**). ^#^*P* < 0.05 and ^##^*P* < 0.01 vs. UCB-MNC group; ^*^P < 0.05 and ^**^P < 0.01 vs. model group

Similarly, the protein level of MMP13 was significantly downregulated in the PRP group and UCB-MNC group, while that of COL-II was upregulated in the UCB-MNC group. Interestingly, p-PI3K and p-AKT were significantly upregulated in the UCB-MNC group, suggesting the involvement of thePI3K/AKT signaling pathway (Fig. 8).

## Discussion

Progressive degeneration of articular cartilages is a pathological benchmark of OA [19]. Articular cartilage is a special type of connective tissue consisting of chondrocytes and extracellular matrix (ECM). Type II collagen is the main structural protein responsible for the formation of ECM network alongside aggregated proteoglycans [20, 21]. Generally, ECM metabolism has a low pace and is regulated by chondrocytes. Cartilage catabolism and anabolism are mainly maintained by matrix metalloproteinases (MMPs) and endogenous tissue inhibitors of metalloproteinases (TIMPs). Matrix metalloproteinase 13 (MMP-13), the key MMP factor, destructs articular cartilage in OA by degrading COL-II [22–24]. Under normal circumstances, a dynamic balance exists between catabolism and anabolism of articular cartilage. Pathological stimuli, nevertheless, trigger inflammatory response of macrophages by the phagocytosis of fragmented chondrocytes. As a result, type A synoviocytes, which are phagocytic and resemble macrophages, become inflamed to release inflammatory factors and MMPs, thus accelerating the decomposition of chondrocytes and the progression of OA [25–27]. Therefore, inflammatory response and degradation of COL-II can be suppressed to protect KOA.

ACLT is a widely accepted procedure to mimic KOA [28]. Joint pain, the primary symptom of KOA, seriously limits the range of motion and normal gaits [29]. Therefore, we graded the Lequesne MG behavioral score in rabbits via assessing the local pain, range of motion, joint swelling and gait changes. The lowest score was observed in the UCB-MNC group, suggesting that injections of UCB-MNCs into knees effectively alleviated the impairment on the functional behaviors in KOA rabbits. KOA is traditionally diagnosed by radiographs, and subchondral osteosclerosis and osteophyte formation are considered as early markers of KOA on CT scans [30]. It is reported that injection of mesenchymal stem cells (MSCs) into knees exerts an analgesic property, and its combination with graphene oxide effectively repairs bone defects, as manifested on CT scans in KOA rabbits [31]. In the present study, micro-CT scanning clearly visualized a significant amelioration in joint stenosis and osteophyte formation in KOA rabbits intervened by UCB-MNCs. Pathologically, well-arranged articular chondrocytes in neat layers are gradually damaged by KOA as the disease progresses [32]. The Mankin scoring system is frequently applied to assess cartilage structure, cellularity, Safranin O staining and tidemark integrity in KOA [33]. Our data revealed the lowest Mankin score in the UCB-MNC group, suggesting that injections of UCB-MNCs into knees effectively alleviated histological changes in KOA rabbits. Growing evidence has supported the essential roles of pro-inflammatory factors and pro-inflammatory signaling pathways in the pathophysiology of OA [34]. IL-1β and TNF-α are two major pro-inflammatory factor involved in the progression of OA in vivo and in vitro [35, 36]. Bone marrow mesenchymal stem cells are reported to reduce local inflammation in the KOA rabbit model through decreasing TNF-α and IL-1β levels in the joint fluid [37]. We consistently examined the elevations of TNF-α and IL-1β levels in synovial fluid of the knee in the model group, and their relative levels were most pronouncedly reduced in UCB-MNC group. Cartilage matrix plays a role in supporting, protecting and nourishing chondrocytes, among which natural collagen fibers can maintain the stability of tissue structures. MMPs are activated after KOA-induced cartilage injuries and distort the structure of the cartilage matrix by degrading type II collagen fibers. Eventually, the cartilage thins, ruptures, or even completely crumbles [38]. Bone marrow mesenchymal stem cell therapy for osteoarthritis yields an effective outcome by downregulating MMP-13 and upregulating COL-II in articular cartilages [39]. Our data also showed that injections of UCB-MNCs into knees significantly downregulated MMP-13 and upregulated COL-II, thus exerting the protective effect on articular cartilages of KOA rabbits.

The PI3K/AKT signaling pathway is well known for its regulatory effect on cartilage degradation by mediating homeostatic and cellular activities [40, 41]. Lu et al. have reported that the activated PI3K/AKT signaling pathway attenuates chondrocyte damage in OA via inhibiting the secretion of MMP13 [42]. Upregulations of p-PI3K and p-AKT were more prominent in the UCB-MNC group than in the model group, suggestive of the activation of the PI3K/AKT signaling pathway in protecting KOA.

There were some limitations in our study. First, we did not profile the safety of knee injections of UCB-MNCs in the treatment of KOA. Second, downstream targets of the PI3K/AKT signaling pathway that are responsible for the therapeutic effect of UCB-MNCs should be further examined. Third, the clinical application of UCB-MNCs to KOA requires further explorations in clinical trials.

## Conclusion

Injections of UCB-MNCs into knees protect the articular cartilage and inhibited the progression of KOA in rabbits by optimizing the local microenvironment at knee joints.

## Data Availability

No datasets were generated or analysed during the current study.
